# P-690. Quantifying The Health Equity-Related Public Health Impact of Influenza Vaccination: A Pilot Study for United Kingdom

**DOI:** 10.1093/ofid/ofae631.886

**Published:** 2025-01-29

**Authors:** Eliana Biundo, Victoria G Soares, Irshaad Jansen, Tomas Mrkvan

**Affiliations:** GSK, Wavre, Brabant Wallon, Belgium; GSK, Wavre, Brabant Wallon, Belgium; GSK, Wavre, Brabant Wallon, Belgium; GSK, Wavre, Brabant Wallon, Belgium

## Abstract

**Background:**

While influenza vaccination is known to positively impact health equity, vaccination recommendations vary by country and may not always optimize equity considerations. For example, while the US recommends vaccination for everyone aged ≥6 months, the UK’s recommendations are limited to adults aged ≥65 years, those aged < 65 years at higher risk, and specific age groups of children. This pilot study measured the health equity-related impact of broader UK vaccination recommendations.

**Figure:**
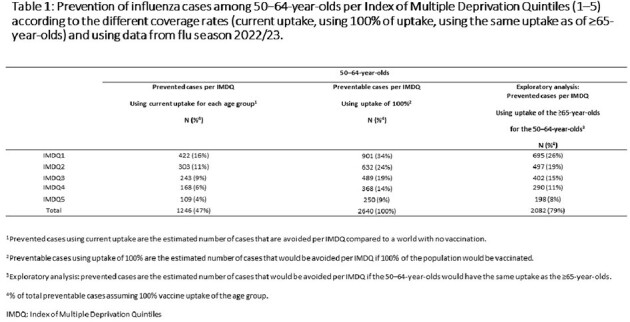

**Methods:**

Data availability determined that this analysis focused on adults aged ≥50 years in England. Health equity impact was assessed by Index of Multiple Deprivation Quintiles (IMDQ) and age (50–64, ≥65 and ≥50 years). Influenza risk and prevented cases were estimated using influenza hospital admission rates, vaccination effectiveness and coverage (flu season 2022/23). An exploratory analysis was conducted assuming extension of UK national guidance to all ≥50-year-olds with equal vaccine uptake in 50–64-year-olds as in ≥65-year-olds.

**Figure:**
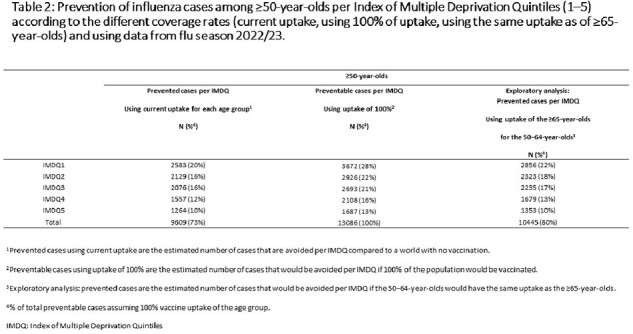

**Results:**

This study estimated vaccination currently prevents ∼80% of all preventable cases in ≥65-year-olds vs. 47% in 50–64-year-olds. Influenza risk is highest in IMDQ1 (most deprived group) across age and vaccination status. Influenza prevention from vaccination of 50–64-year-olds (coverage ∼50%) in England is greatest in IMDQ1 (16% of all preventable cases prevented vs. 4% in IMDQ5, least deprived) (**Table 1**). Assuming extension of UK national guidance to all ≥50-year-olds with similar coverage to ≥65-year-olds (∼80%) may prevent ∼26% of preventable cases in IMDQ1 and ∼8% in IMDQ5 (**Tables 1−3**).

**Figure:**
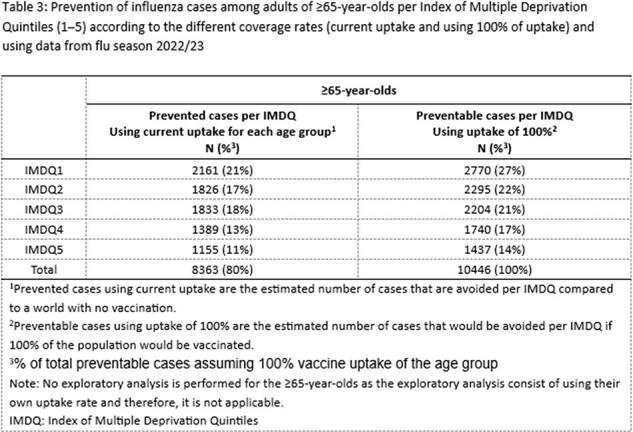

**Conclusion:**

Expanding influenza vaccination to other age groups can help increase health equity by improved influenza prevention. Greater vaccination coverage would enable the highest reduction in cases, especially in the most socially disadvantaged groups.

**Funding:** GSK

**Disclosures:**

**Eliana Biundo, MSc**, GSK: Employee|GSK: Stocks/Bonds (Private Company) **Victoria G. Soares, MBA**, GSK: Advisor/consultant **Irshaad Jansen, MSc**, GSK: Employee|GSK: Stocks/Bonds (Private Company) **Tomas Mrkvan, PhD**, GSK: Employee|GSK: Stocks/Bonds (Private Company)

